# Socio-cultural factors for breastfeeding cessation and their relationship with child diarrhoea in the rural high-altitude Peruvian Andes – a qualitative study

**DOI:** 10.1186/s12939-021-01505-3

**Published:** 2021-07-16

**Authors:** Néstor Nuño Martínez, Jordyn Wallenborn, Daniel Mäusezahl, Stella M. Hartinger, Joan Muela Ribera

**Affiliations:** 1 Department of Public Health and Epidemiology, Swiss Tropical and Public Health Institute, P.O. Box CH-4002, Socinstrasse 57, Basel, Switzerland; 2 University of Basel, P.O. Box CH-4001, Petersplatz 1, Basel, Switzerland; 3 Universidad Peruana Cayetano Heredia, Av. Honorio Delgado 430, urb. Ingeniería, S.M.P, Lima, Peru; 4 Partners for Applied Social Sciences (Pass-International), Baal 58, Tessenderlo, 3980 Belgium; 5 Universitat Rovira i Virgili, Avinguda Catalunya 35, Tarragona, 43005 Spain

**Keywords:** Breastfeeding, Child, Child diarrhoea, Socio-cultural factors, Female, Local explanatory models, Peer-counselling, Pregnancy, Social norms, Peru, Lactancia, Niño, Diarrea infantil, Factores socioculturales, Mujeres, Modelos explicativos locales, Consejería entre pares, Embarazo, Normas sociales, Perú

## Abstract

**Background:**

In some areas of the world, breast milk is seen as a potential source of child diarrhoea. While this belief has been explored in African and Southeast Asian countries, it remains vastly understudied in Latin American contexts. We investigate socio-cultural factors contributing to breastfeeding cessation in rural high-altitude populations of the Peruvian Andes. The role of socio- cultural factors in the local explanatory model of child diarrhoea, and whether these perceptions were integrated in the local healthcare system were assessed.

**Methods:**

Within the framework of a randomised controlled trial, we conducted semi-structured interviews with 40 mothers and 15 health personnel from local healthcare centres involved in the trial.

**Results:**

Cultural beliefs on breastfeeding cessation included the perception that breast milk turned into “blood” after six months and that breastfeeding caused child diarrhoea. We identified eight local types of child diarrhoea, and women linked six of them with breastfeeding practices. “Infection” was the only diarrhoea mothers linked to hygiene and the germ disease concept and perceived as treatable through drug therapy. Women believed that other types of diarrhoea could not be treated within the formal healthcare sector. Interviews with health personnel revealed no protocol for, or consensus about, the integration of the local explanatory model of child diarrhoea in local healthcare and service provision.

**Conclusions:**

The local explanatory model in rural Andean Peru connected breastfeeding with child diarrhoeas. Cultural beliefs regarding diarrhoea management may increase home treatments, even in cases of severe diarrhoeal episodes. Future national breastfeeding support programmes should promote peer-counselling approaches to reduce negative attitudes towards breastfeeding and health practitioners. Local explanatory models should be incorporated into provincial and regional strategies for child diarrhoea management to promote equity in health and improve provider-patient relationships.

## Resumen

**Antecedentes:**

En diferentes partes del mundo, la leche materna es percibida como una fuente potencial de diarreas infantiles. Mientras que estas creencias se han estudiado en África o el Sudeste Asiático, su análisis en el contexto latinoamericano es limitado. Esta investigación se centra en el estudio de los factores culturales que contribuyen al cese de la lactancia materna en poblaciones rurales de los Andes peruanos. Al mismo tiempo, también se analiza el papel de estos factores culturales en el modelo explicativo local de diarreas infantiles y la integración de las creencias en los servicios locales de salud.

**Métodos:**

Dentro de un ensayo clínico aleatorizado, se llevaron a cabo entrevistas semi-estructuradas con 40 mujeres y 15 trabajadores de salud de centros participantes en el ensayo.

**Resultados:**

Las creencias culturales en torno a la lactancia maternal incluían la percepción de que la leche materna se convierten en “sangre” a los seis meses del parto y que la leche materna causaba diarreas en los niños lactantes. Identificamos ocho tipos de diarreas locales, seis de las cuales fueron asociadas con la lactancia por las madres participantes. “Infección” resultó ser el único tipo de diarrea que las madres asociaron con los principios de la higiene y la teoría microbiana de la enfermedad y percibían como curable por medio de medicamentos. Las mujeres creían que el resto de diarreas locales no podían ser tratadas dentro del sistema de salud. Las entrevistas con el personal sanitario indicaron una falta de protocolos y consenso sobre cómo integrar el sistema de creencias locales en torno a las diarreas infantiles en los servicios de salud.

**Conclusiones:**

El modelo explicativo local en zonas rurales de los Andes peruanos asocia la lactancia con las diarreas infantiles. Estas creencias culturales dan lugar a prácticas de manejo de diarreas infantiles que pueden incrementar los tratamientos domiciliarios, incluso en episodios de diarrea severos. Futuros programas nacionales de apoyo a la lactancia materna deben promover la consejería y apoyo de pares y profesionales de salud con la finalidad de reducir las actitudes negativas hacia la lactancia materna y el personal de salud. Los modelos explicativos locales de las diarreas infantiles deberían incorporarse a las estrategias provinciales y regionales con la finalidad de promover la equidad en salud y mejorar las relaciones médico-paciente.

## Background

Diarrhoea is one of the leading causes of morbidity and mortality in children under 5 years. In 2016, there were 446,000 deaths due to diarrhoea among children under 5 years in low- and middle-income countries (LMIC) [1]. Significant levels of diarrhoea infections are strongly correlated with poor housing, hygiene, and sanitation conditions. Recurrent diarrhoea episodes can produce acute malnutrition and increase risk of other serious diseases such as respiratory infections, measles, and pneumonia [2–5]. The co-occurrence of diarrhoea and acute malnutrition at young ages can lead to significant long-term adverse effects in adulthood, such as non-communicable disease or reduced economic productivity [6–8].

Patterns of distress, perceptions on causes and treatment options and practices in dealing with child diarrhoea exist cross-culturally and are generally referred to as local explanatory models [9, 10]. For example, many local types of child diarrhoea are not commonly linked to hygiene and the germ disease concept, instead they are said to originate from environmental or supernatural events [10–12]. In addition, local concepts are typically present and most widely accepted in locations where education levels are low, healthcare systems are weak and traditional medicine or elder family/community members are respected sources of information [13–16]. Local beliefs and concepts regarding causes of child diarrhoea are complex and, in some cases, contradictory. For example, in Tanzania, some ethnic groups consider diarrhoea a life-threatening ailment while in Nigeria or Thailand, other groups seen it as a normal sign of growth and development [16–18]. These perceptions also include interpretations regarding diarrhoea management practices. In some areas of rural Vietnam, mothers consider breastfeeding beneficial during diarrhoea episodes [15]. Yet, in rural Thailand, mothers believe that breastfeeding during diarrhoea episodes worsen the illness [16]. Similarly, in Pakistan, India, and Sri Lanka, some ethnic groups believe that breast milk can be contaminated through witchcraft, a new pregnancy, a maternal illness, or exposure to environmental factors [19, 20].

Breastfeeding is widely recognised as a cost-effective practice that prevents infections and diarrhoea during infancy, and reduces risk of dehydration, severe disease progression and disease duration [21–24]. In fact, estimates suggest that an increase in breastfeeding levels worldwide would prevent almost one million annual deaths in children [8]. The World Health Organization (WHO) recommends exclusive breastfeeding for the first six months of life, followed by complementary breastfeeding up to 2 years of age or beyond [25]. Despite the well-known benefits of breastfeeding, about 63 % of children under six months of age are not exclusively breastfed in LMIC), and rates of breastfeeding cessation increase exponentially after six months of age [8]. The majority of research to date investigates socio-demographic factors related with breastfeeding cessation (e.g., education, maternal depression, previous breastfeeding experiences) [26–30]; however, cultural beliefs may also influence breastfeeding practices.

Various studies around the world examined links between socio-cultural factors for breastfeeding cessation and local explanatory models of child diarrhoea [16, 20], but little is known about these perceptions in the rural high-altitude Peruvian Andes. Despite being a middle-income country, Peru is characterised by large social and health disparities. 29 % of children under 5 years of age in the lowest poverty quintile suffer from stunting and 39 % from anaemia compared to 4 and 24 % in the richest quintile [31]. According to the 2016 National Demographic and Family Survey, Peru has a high 2-week prevalence of diarrhoea (11 %) in children < 5 years of age. In addition, 69 % of children < 6 months were exclusively breastfeed, with a median duration of 4.1 months [32]. Previous studies conducted in high-altitude Andean settings show that breastfeeding practices follow culture-specific beliefs, suggesting that mothers may cease exclusive breastfeeding temporarily or permanently according to local illness explanatory models [33–36].

Considering the impacts of cultural beliefs on breastfeeding practices and child diarrhoea management in Andean settings, our study: (i) identifies socio-cultural factors for breastfeeding cessation and their connection with the local explanatory model of child diarrhoea in rural high-altitude Andean Peru and, (ii) explores whether these perceptions and beliefs are integrated in the local healthcare and service provision.

## Methods

### Study site and participants

Women participants were identified from a community-randomised controlled trial (henceforth referred to as the “IHIP-2”) [37] evaluating the impact of health and early child development home-based interventions in 82 rural communities. IHIP-2 took place in San Marcos and Cajabamba, two rural high-altitude resource-limited provinces situated in Northern Andean Peru (Cajamarca). IHIP-2 was conducted among households with children < 18 months that were not enrolled in the national early child development (ECD) programme and belonged to one of the four trial arms with either (i) an improved biomass cookstove, or (ii) an ECD intervention, (iii) both interventions or (iv) none (control). The majority of families participating in IHIP-2 lived in adobe households and practised subsistence agriculture (i.e. agriculture outputs are used to meet familial needs). Breastfeeding counselling in communities was mostly provided by health personnel at healthcare centres. A full description of the site, participants and IHIP-2 design is found in Hartinger et al. [37]. We also invited health personnel from six local healthcare centres involved in IHIP-2 trial to participate in the study.

### Study design

Our qualitative study was conducted between June and December 2016. We approached 10 women from each of the four study arms of IHIP-2 (40 in total) and invited them to carry out semi-structured interviews. No specific criteria (e.g., saturation) were applied to determine the final sample size. The inclusion criteria for women were being a mother or female caretaker of a child participating in the IHIP-2 trial. One mother refused to participate lacking the support of her husband and was replaced. The interviews took place in women´s households. The objective was to identify socio-cultural factors regarding breastfeeding cessation and investigate their connection with the local explanatory model of child diarrhoea.

We complemented mother perceptions with the views of local health personnel. We invited fifteen health personnel by convenience and their time availabilities to carry out semi-structured interviews. To identify health personnel participants, we used the chain-referral sampling technique. This sampling technique consists of an initial contact with a small group of people relevant to the research topic, and then draws on the networks of those people to establish new contacts [38]. Sample size for interviewing health personnel was determined by saturation, i.e., recruitment stopped when no new relevant data emerged on the research topic [39]. Two health practitioners were not able to participate due to time constraints and were replaced. The interviews took place at healthcare centres.

Interview guides and topics discussed were identical for mothers and for health personnel and were based on previous studies in LMIC that qualitatively explored the relationships between socio-cultural factors for breastfeeding cessation and child diarrhoea [17, 39]. Interview guides for women and health personnel were piloted with two participants from each group. All study participants, mothers and health personnel, were personally invited by the study team. Interviews were only conducted once. All interviews were carried out in Spanish, tape-recorded, and had an approximate duration of 1 h. No extra field notes were taken during interviews. All interviews with women and health personnel were conducted one-on-one by a medical anthropologist (NN) who was also involved in the IHIP-2 trial. This characteristic was reported to all study participants. Only NN was present during the interviews with study participants. NN established a previous relationship with women participants (informal contacts during IHIP-2 home-visits) prior to study commencement. Women were informed that the research sought to understand their knowledge about and experiences with childhood diseases in the area. Health personnel were not contacted prior to the interview. For them, the main study goal presented was to understand their knowledge, attitudes and practices of local perceptions of childhood illnesses.

Our study complies with the guidelines of the Consolidated Criteria for Reporting Qualitative Research (COREQ). The COREQ is a 32-item checklist developed to facilitate the understanding of the methods, data analysis and results of qualitative studies [40].

### Data analysis

Women and health personnel interviews were transcribed verbatim using Express Scribe transcription software (NCH software, Canberra, Australia). Transcripts were not returned to participants for comments or corrections. NN then conducted a manual standard content/thematic analysis for women and health personnel interviews [39]. First, information was read, edited, derived in basic themes and organised into categories according to the designated analytical guides (e.g. socio-cultural factors for breastfeeding cessation, local types of child diarrhoea, diarrhoea management recommendations and provider-patient relationships). Secondly, NN and another anthropologist (JM) compared information among categories to identify different opinions among participants. Finally, a general interpretation for each category was derived from the comparative analysis and appropriate quotes were selected to illustrate findings. Any new themes that emerged during the process were included in the analysis. The local explanatory model of child diarrhoea was developed inductively applying the principles of the grounded theory. The transcriptions and coding tree can be provided upon request. Neither women nor health personnel were invited to provide feedback on the findings. Baseline information from IHIP-2 was used to describe characteristics of women participants. Data were entered in Census and Survey Processing System 6.3 (United States Census Bureau, USA) and then exported to STATA 15.0 Statistical software (STATA CORP. College Station, Texas, USA) for analysis. We performed a descriptive analysis using means, medians, and percentages [37].

## Results

### Women’s characteristics and breastfeeding practices

The majority of women participants lived in households with adobe walls, earthen floors, tiled roofs, and had electricity for lighting. Households had an average of 2.5 rooms (SD = 1.0) and the mean number of inhabitants per household was 4.7 (SD = 1.4). Most women were married or had a civil partnership (85 %), and their mean age was 28 years (SD = 8.1). Women average years of schooling was 6.0 (SD = 3.8), and, housework (97 %) and self-employment (55 %) were their primary and secondary occupations. The median number of children was 2 (IQR = 1, 3). At the time of the study, mean age of children participating in IHIP-2 was 1.8 years (SD = 0.5) and 55 % of women (*N* = 22) had ceased breastfeeding. Among mothers who ceased breastfeeding, 85 % (*N* = 18) declared having done before their child reached 2 years of age. Households were evenly distributed across different poverty quintiles (Table 1).

**Table 1:** Household, demographic, and wealth characteristics of women participants and their children

	N	N (%)/Mean (SD)/Median (IQR)
*Household characteristics*	40	
Adobe wall type		36 (90.0)
Earthen floor type		35 (87.5)
Roof tile type		33 (82.5)
Electricity		31 (77.5)
Number of rooms		2.5 (1.0)
*Demographic characteristics*	40	
Number of inhabitants per household		4.7 (1.4)
Civil status (married/civil partnership)		34 (85.0)
Maternal age (years)		28.1 (8.1)
Maternal years of schooling^a^		6.0 (3.8)
Primary maternal occupation (housework)		39 (97.5)
Secondary maternal occupation (self-employed)		22 (55.0)
Number of children per woman		2 (1, 3)
Child age (years)^b^		1.8 (0.5)
Breastfeeding cessation^b^		22 (55.0)
Before the child reached two years	21	18 (85.7)
*Wealth classification*^c^	40	
1. Quintile (lowest)		8 (20.0)
2. Quintile		8 (20.0)
3. Quintile		8 (20.0)
4. Quintile		9 (22.5)
5. Quintile (highest)		7 (17.5)

^a^Includes primary (1-6 years) and secondary (7-12 years) education. For higher degrees, we considered: higher (not university) education not completed (12.5 years), higher (not-university) education completed (14 years), university education not completed (13.5 years) and university education completed (16 years)

^b^Information collected at the time of study, six months after IHIP-2 baseline. It refers to mothers/children participating in IHIP-2

^c^Calculated using the nationally validated Young Lives wealth index [41]

### Socio-cultural factors for breastfeeding cessation

All women reported receiving regular breastfeeding counselling in local healthcare centres. In general, they indicated that breastfeeding helped children to grow up stronger and develop their cognitive potential. Women mentioned four major reasons for breastfeeding cessation based on biological, and social, and socio-cultural factors. The first reason was low breast milk supply. Women believed that this was caused by a high number of pregnancies within a short period of time. This perception generally originated from exchanges with relatives and friends and the doctor’s advice: “*My breasts hurt since my last child was born, and I almost had no breastmilk. The doctor told me that breast milk was important and I should continue giving the formula milk to my baby*” (Interview with Nekane, July 4, 2016). Time commitment was mentioned as another reason for breastfeeding cessation. Children’s continuous demands to be breastfed made it difficult to complete daily chores, leading to distress and fatigue:

*“I ceased breastfeeding when my child was about a year and four months old. He did not want to eat food and wanted to breastfeed at any time. I took him to harvest Alfalfa [Medicago sativa] and he was always asking for breastfeeding. It was annoying because he did not let me work”* (Interview with Matilda, June 16, 2016).

Thirdly, some women believed that the quality of breast milk started declining six months after childbirth, and then loses its nutritional value and becomes a drink made of blood: “Breastfeeding is good until the sixth month, but never after. This is because breast milk becomes *chuvia* [*soft*], like water. It is no longer dense” (Interview with Ana, September 20, 2016). Some of these women associated changes in the structure and composition of breast milk with their own diet. A diet rich in legumes and animal protein would lead to good-quality breast milk. In contrast, excessive consumption of cereals, rice, and potatoes would make the breast milk *chuvia*. In general, women found that breastfeeding represented a communication channel between mothers and children. This perception had beneficial connotations such as the transmission of positive emotions and the strengthening of mother-child bonds. However, mothers also considered that breast milk could cause child diarrhoea, and thus, lead to breastfeeding cessation:

*“Breastfeeding was not good for her* [my child]. *She was always ill. She seemed to recover but then it* [the disease] *came again. People told me to cease breastfeeding, that it was the problem [for her repeated illnesses]. People also explained to me that if adults are ill then the disease is transmitted to children through breastfeeding* […] *Breast milk can produce many types of diarrhoeas”* (Interview with Laura, July 7, 2016).

### Breastfeeding and the local explanatory model of child diarrhoea

Overall, women participants indicated that breastfeeding was a potential cause of child diarrhoea when it was carried out within certain contexts and periods:

*“Children get everything from breast milk. When they are sick, we* [mothers] *have to take the medicine too to help them recover* […] *Even if mothers are sick, children can get sick from the breast milk* […] *In addition, what mothers eat regularly can also affect children’s health if they breastfeed” (Interview with Marina, August 8, 2016).*

According to mothers, child diarrhoea in the local illness model was caused by either (i) “hot milk” (*leche soledada*); (ii) “cold milk” (*leche fria*); (iii) “hidden heat” (*calor recogido*); (iv) “dull milk” (*empacho*); (v) “choleric milk” (*cólera*); or (vi) “spoiled milk” (*leche mala*).

Regular breast milk would transform into “hot milk” if directly exposed to the sun and the consumption of “hot milk” produced an odorous yellowish or whitish abundant diarrhoea and persistent vomiting. Mothers defined “cold milk” (also called *chiriaiti*) as the opposite of “hot milk”, noting that breast milk became cold when residing in a cold environment or coming into contact with a cold substance (e.g., water). This type of breast milk, they claimed, produced mucosal greenish diarrhoea and vomiting. Likewise, mothers linked the occurrence of “hidden heat” to the time of year when temperature fluctuations were common. They described this ailment as a combination of “cold milk” and “hot milk”:

*“My first daughter was always ill and I did not know the reason. At the healthcare centre, the personnel told me it was diarrhoea. Thus, I gave her medicines but they never worked. Then, I went to the pharmacy and what they gave me did not work either. She was always ill. That is the reason why I tried a homemade treatment that people told me to use. It was to treat a disease between the “hot” and the “cold”. We call it “hidden heat””* (Interview with Marina, August 8, 2016).

According to women, the fourth type of harmful breast milk, “dull milk”, was closely related to nutrition. A diet mainly composed of carbohydrates would make the breast milk thicker, generating stomachaches and foamy stools in children. In contrast, “choleric milk” was thought to have a psychosomatic origin, as its appearance was linked with emotional distress:

*“Imagine that today I went to a place and people start saying horrible things about my family or me, like that my husband is alcoholic. Then I got angry and I breastfeed my child. All that irritation and negative feelings pass to my child, who becomes sick. Children can die with this disease, because when they have “choleric milk” they vomit, have diarrhoea, their skin becomes pale, and their tummy is swollen”* (Interview with Lucrecia, July 7 2016).

Women described diarrhoea produced by “choleric milk” as yellowish and with profuse passing of stool. In order to distinguish “hot milk” from “choleric milk” (both had a similar appearance), some women recommended rubbing an egg against the child’s extremities and then emptying it into a glass to see the “origin” of the disease. According to mothers, this was also practiced to identify other pathologies included in the local illness model, such as the “evil eye”. “Choleric milk” is particularly dangerous for children as it made them thinner and causes their nails to blacken. Mothers emphasised that “choleric milk’” was potentially fatal if treated at healthcare centres:

*“When children have “choleric milk” we do not go to healthcare centres. When it is too strong, doctors give injections that kill the child. At healthcare facilities, there is no medicine for “choleric milk”. What they use is worthless. However, when children take our herbs* [homemade beverages] *or the anti-choleric syrup, they recover quickly” (Interview with Angela, July 19, 2016).*

Finally, women asserted that breastmilk got spoiled (“spoiled milk”) with a new pregnancy while still breastfeeding a toddler: “It is as if the other baby were jealous. This ailment delays pregnancy and makes children sick” (Interview with Teresa, August 15, 2016). “Spoiled milk” implied possible adverse pregnancy outcomes as well as diarrhoea and vomiting in the nursed child. Child diarrhoea produced by “spoiled milk” would have a liquid texture and different colours (e.g., yellowish, greenish). Some stated that “spoiled milk” only appeared when the new child was of a different sex than the currently breastfed one.

Women also describe different treatments and methods of prevention for each local type of child diarrhoea. To prevent “hot milk”, mothers recommended expressing some breast milk before giving the breast to the child or avoiding breastfeeding under the sun. Mothers believed that diarrhoea produced by “hot milk” could only be treated with “cold” herbs. They also endorsed rubbing the child’s stomach and forehead with herbs such as the sacred leaf (*Piper auritum*) or chicory (*Chicorium intybus*). For preventing “cold milk”, mothers suggested drinking hot beverages before breastfeeding. Remedies included the use of “hot” herbs or homemade treatments such as rubbing children’s upper and lower extremities with hot water, massaging the chest with eucalyptus extract, or eating fried chicken fat. To treat “hidden heat”, mothers prepared beverages combining “cold” and “hot” ingredients. To prevent “dull milk”, mothers recommended consuming purgative herbs used for stomach cleansing (e.g., *sen* (*cassia acutifolia*) or olive oil). Treatments for “dull milk” included tipping children upside-down and patting them on the back to expel gases. Mothers indicated that the only way to prevent “choleric milk” was to avoid negative thoughts. Treatments included the use of “cold” herbs and the so-called anti-choleric syrups or pills. They were sold in pharmacies and grocery stores, and the price per dose varied between 10 centavos and one Peruvian sol (0.03–0.30 US$). Finally, mothers argued that the only treatment and solution for “spoiled milk” was breastfeeding cessation. As a general remark, women emphasised that none of these local types of child diarrhoea could not be treated with medical drugs. Table 2 describes the local explanatory model of child diarrhoea and its relationship with breastfeeding practices.

**Table 2:** Local types of child diarrhoea associated to breastfeeding in rural Andean Peru

Local name	Perceived cause	Symptoms	Diarrhoea-specific perceptions			Preventive practices and beliefs	Treatment preferences
			Colour	Texture	Quantity		
"Hot milk”	Hot	- Diarrhoea	White or yellow	Pasty	Abundant	- Avoid breastfeeding in/after sun exposure	- “Cold” herbs
		- Fever					- Rubbings
		- Vomits					
“Cold milk”^a^	Cold	- Diarrhoea	Green	Mucous	Irregular	- Avoid breastfeeding in/after sun exposure	- “Hot” herbs
		- Vomits				- Consumption of hot beverages	- Rubbings
“Hidden heat”	Hot and Cold	- Diarrhoea	-	-	-	-	- “Hot” herbs/“Cold” herbs
		- Vomits					
“Dull milk”	Personal diet	- Abdominal pains	-	-	-	- Back slapping	- Back slapping
		- Constipation				- Stomach cleaning	- Stomach cleaning
		- Foaming stools					
“Choleric milk”	Emotional status	- Diarrhoea	Yellow	Pasty	Abundant	- Avoid negative thoughts	- “Cold” herbs
		- Vomits					- Rubbings
							- Anti-choleric
“Spoiled milk”	New pregnancy	- Diarrhoea	Yellow or green	Liquid	-	-	- breastfeeding cessation
		- Vomits					

^a^Also called *chiriaiti*

Women participants linked the previously described set of locally defined types of child diarrhoea with two other ailments included in the local illness model: (i) “infection” and (ii) “fright sicknesses” (*susto*). “Infection” was a diarrhoea caused by bacteria, bugs (parasites and insects), and dirt that lived on the ground. Mothers described the stools associated with this ailment as having a pasty yellowish texture. To treat “infection” diarrhoea, mothers recommended the use of “cold” herbs and antibiotics such as Trimethoprim Sulfamethoxazole. Antibiotic treatment (generally obtained in pharmacies without medical prescription) was though preferred due to the belief that children would heal faster. Mothers asserted breastfeeding was safe when a child had “infection” diarrhoea. For this reason, some also consumed antibiotics themselves during their child’s diarrhoea, as they thought medicine would be passed to the child through the breast milk and accelerate the recovery. Overall, “infection” was the only child diarrhoea that women perceived as treatable with western medicines.

“Fright sickness” (*pachachari*) was believed to have a psychosomatic origin: “It is when something scares our children. When they get the *pachachari*, they no longer eat nor sleep and they have hefty diarrhoea” (Interview with Agueda, August 13, 2016). “Fright sickness” make children cry continuously and “makes them thinner”. Mothers described this type of diarrhoea as liquid and profuse (e.g., a texture similar to water) and with several colours (e.g., yellow, white, green). To treat “fright sickness”, families carried out rituals called *limpias*. These consisted in rubbing the child’s body with a special white stone (*lumbre*) at midnight on Tuesdays or Fridays. In order to be effective, the ritual had to be repeated over 3 days. It is also possible to perform the *limpia* ritual at noon. Subsequently, the *lumbre* had to be thrown into a fire. When the fire went out, an image of the entity that had “scared” the child would appear in the ashes. Mothers emphasised the importance of hiding or removing these ashes as other people might use them to invoke the child’s soul and make black magic rituals. The *lumbre* stones were normally sold in pharmacies and grocery stores. Mothers did not mention that breastfeeding would worsen child diarrhoea when a child had “ fright sickness”.

### Integrating the local illness model into healthcare and service provision

The majority of health personnel recognised the existence of a local wealth of beliefs and interpretations concerning child diarrhoea. However, they stated mothers or caretakers generally did not talk about these pathologies during medical consultations:

*“Today, we do not hear too much of them* [local beliefs]. *In earlier times, mothers said their children were malnourished because they had been scared. However, those beliefs are already modified. Maybe some families still believe those things, but we no longer hear of them” (Interview with the obstetrician Facunda, October 20, 2016).*

Overall, interviews showed that local causal models and traditional beliefs in general were rarely considered in routine medical care. In addition, there was also no consensus among care providers on how to best accommodate them. The most experienced health personnel showed the greatest willingness to incorporate mother´s beliefs concerning child diarrhoea in routine medical care. On the contrary, the less experienced healthcare workers believed that families should modify their attitudes. Some women participants indicated that negative attitudes of health personnel towards local beliefs stopped them from using healthcare facilities when their children had a locally defined type of child diarrhoea:

*“Last year, my child had fever and sores in his mouth, so I went to the healthcare centre. They gave him syrups but nothing happened. A neighbour told me to look for a special herb in order to prepare a beverage. My child drank it and recovered quickly. I think he had a stomach fever because his mouth was very hot. However, at the healthcare centre they told me that it was impossible, that it [stomach fever] did not exist” (Interview with Paula, August 16, 2016).*

Health personnel identified chronic lack of personnel and high staff turnover as the main reasons for worsening relationships with rural patients. They acknowledge that conflict situations were common, especially when medical trainees from urban Peru came to provinces to do their mandatory internship (The Rural and Urban Marginal Health Service (SERUMS) programme). Some health practitioners stated that, sometimes, participants of the SERUMS programme completely dismissed traditional concepts and beliefs during doctor-patient interactions:

*“They do not tell us about them anymore* [local beliefs] *because some personnel says, “hey, this is not right”. Some personnel explain things kindly to mothers. Others, however, harshly dismiss traditional viewpoints right in front of them. That is what makes mothers not want to come back. Those are especially the fresh trainees, - the serumes* [personnel from the SERUM programme]. *They come from the cities and have their own ideas. They do not care about what people think here. They come, do their service, and then leave again” (Interview with the nurse Patricia, November 2, 2016).*

## Discussion

Our study describes socio-cultural factors for breastfeeding cessation and their relationship to the local explanatory model of child diarrhoea in rural high-altitude Andean Peru. Women participants mentioned four major reasons for breastfeeding cessation based on biological, social, and socio-cultural factors. One biological factor was low breast milk supply, which women believed that it was caused by a high number of pregnancies within a short time period. Another social factor included time commitment, as children’s continuous demands to be breastfed made it difficult for women to complete daily chores. Cultural beliefs included the perception that the quality of breast milk declined six months after childbirth and that breastfeeding caused child diarrhoea. Mothers considered breastfeeding important for healthy development of new-born children. However, they also stated that breastfeeding might produce diarrhoea within certain contexts and periods (e.g., while exposed to hot/cold environments). Mothers identified eight local types of diarrhoea, and connected six of them with breastfeeding. Among these types of child diarrhoea, mothers identified only one (“infection”) with hygiene and the germ disease concept, and perceived it as treatable through drug therapy. Interviews with mothers and local health personnel demonstrated a lack of integration of these beliefs within the local healthcare and service provision as well as a strong refusal among mothers to get treatment for all but one type of diarrhoea at healthcare centres.

### Socio-cultural factors of breastfeeding

Our study provides evidence that the “hot and cold” theory is culturally integrated into mothers’ perceptions of diarrhoea aetiology. The “hot and cold” theory describes the balance between physical and psychosocial states [10, 42, 43]. Previous research from other Andean settings present similar findings. In a study assessing indigenous and public health infant feeding recommendations in rural high-altitude Southern Andean Peru, mothers argued that breast milk was a “cold” medicine used to cure “hot” ailments such as “hot” fevers, backache or eye infections. However, they also perceived that an unbalanced diet and exposure to various environmental factors like heat, cold or wind contaminated breast milk. Expressing some breast milk before nursing was seen as a preventive practice, but mothers declared that extracted breast milk might cause illness when exposed to the sun [34]. In another study assessing health-seeking behaviours in rural high-altitude Andean Peru and Bolivia, mothers reported that diarrhoeas could be caused by breast milk from a mother experiencing emotional distress (*cólera*) [35]. Finally, a study conducted in the Southern Puno region (Peru) described how Andean women had to dress warmly while breastfeeding to prevent the transmission of “cold”-related diarrhoeas to their children [44]. Similar perceptions are observed in other LMIC. In India, Tanzania and Thailand, mothers believe their emotional and health state is transmitted through breast milk [16, 17, 45].

Evidence suggests that breastfeeding is an intersubjective behaviour that is articulated differently between cultural, social, economic, biological, psychological, symbolic, and political dimensions [46–48]. In all societies, breastfeeding is used as a means for managing emotions, reinforcing social norms, defining cultural images and identities, and strengthening mother-child relationships [49–52]. For example, in rural Mexico breastfeeding becomes less accepted (and even reproached) when the child is breastfed more than a year or when it starts growing teeth. Moreover, women perceive breastfeeding in public as a practice of the poor and of rural and indigenous women [53].

### Health-seeking behaviours for treating child diarrhoea

We found that mothers in our study did not consider breastfeeding useful to prevent or mitigate local types of child diarrhoea; therefore, other treatment options (herbal medicine) were used. This contradicts the biomedical notion that breastfeeding prevents diarrhoea infections and wasting in children. Overall, mothers noted that breastfeeding was only useful for treating one type of diarrhoea linked to hygiene and the germ disease concept (“infection”). They also reported using antibiotics to treat this type of diarrhoea due to the perception that the antibiotics cured diarrhoea faster. Ideas about the efficacy and effectiveness of medicines have unique cultural dimensions. In Southeast Asia and Central and South America, some people doubt the efficacy of oral re-hydration solution for treating diarrhoea because its appearance does not resemble medicine [12, 54, 55]. Studies highlight that antibiotics are commonly used with or without medical prescription for treating non-antibiotic-associated child diarrhoea in Peru and other LMIC [56, 57]. This practice is not recommended by the WHO and has negative effects on anti-microbial resistance and the development of infant's gut microbial [58]. In our setting, self-medication with antibiotics was a common practice to treat “infection” diarrhoea and even women consumed antibiotics themselves at the same time. They believed that healing effects of antibiotics were transmitted to their children through breast milk, reducing recovering times. Similar behaviours are reported in other LMIC such as Thailand [16].

### Local beliefs and healthcare and service provision

The incorporation of local beliefs and traditional medicine practices to the biomedical system is critical for delivering adequate quality of care and improving health outcomes [59–61]. We found that local causal models and traditional beliefs were rarely considered in routine medical care at local healthcare centres. In addition, we found that some mothers had negative attitudes towards health personnel, which may cause unintended harmful effects by reducing health seeking behaviours that could lead to an inappropriate management of bacterial diarrhoeas. Cultural competence education for healthcare professionals gained relevance in recent years [59], and different studies emphasise that South American illness explanatory models can be easily incorporated into local healthcare and service provision [35, 62–65]. However, systematic reviews of interventions applying cultural competence education approaches only found moderate improvements in health provider outcomes and healthcare access and weak improvements in patient-provider relationships. The heterogeneity of intervention strategies, measures and outcomes are the main limitations described [66, 67].

### Practice implications

We believe our study is especially interesting for health professionals, policymakers and researchers, as it describes practices and beliefs that could be incorporated into programmes currently underway in rural high-altitude Andean Peru. The Peruvian national ECD programme (PNCM) was launched in 2012, to improve the cognitive, social, and emotional development of infants < 36 months living in poverty. In rural areas, the PNCM provides home-visiting interventions consisting in weekly play-oriented activities and monthly communal meetings carried out by trained women living in the communities (mother facilitators (MFs)). In 2018, a new decree fostered the promotion of breastfeeding in the PNCM by encouraging breastfeeding practices and counselling pregnant women for immediate breastfeeding after childbirth. The decree specifically focused on technical aspects of breastfeeding (e.g., position, timing), but other structural and socio-cultural dimensions are not discussed.

All mothers in our study received breastfeeding counselling at healthcare facilities. Yet, we found that cultural perceptions and breastfeeding practices prevailed, and premature breastfeeding cessation was common. The approach used to promote breastfeeding can partially explain the persistence of this unhealthy practice: some authors argue that promoting specific ideas and principles regarding breastfeeding practices such as WHO’s recommendations without considering the local context may generate blurred interpretations and decrease the effectiveness of breastfeeding interventions [47, 48, 68, 69]. In this regard, peer-counselling breastfeeding programmes using trained women living in the communities proved to be effective to improve rates of breastfeeding initiation, duration, and exclusivity and reduce child diarrhoea incidence in rural settings [70, 71]. In addition, these programmes are cost-effective [72, 73] and have long-lasting effects [74]. The main reason behind the success of peer-counselling breastfeeding programmes is community engagement, as mothers highly appreciate to have someone from their own community to help them with their breastfeeding problems [75, 76]. Despite PNCM’s component on breastfeeding, it does not currently follow a peer-counselling approach. The structure of PNCM’s weekly visits and the role of MFs could facilitate the inclusion of peer-counselling as local rural populations are already involved in regular MFs visits. This approach would also improve the cultural competence of health personnel in the matter if they are partly embedded in the process and promote peer-counselling principles in the interaction with the patients. Achieving this goal would require important efforts and active dialogues between institutional and political levels [62].

### Limitations

Our qualitative study design does not allow for causal inference, or for an epidemiological interpretation of cultural breastfeeding habits and child diarrhoea, nor on the local management of child diarrhoea episodes. In addition, we did not investigate the impact of social networks on the preservation and diffusion of cultural beliefs such as that breast milk became “blood” six months after childbirth, although some of the interview extracts showed that these dimensions may be important. Based on our findings, some potential topics for improving the cultural competence of health personnel in our setting can be identified. However, in order to propose specific actions/guidelines, additional studies should be conducted. Finally, the enrolment of participants with similar socio-economic and educational background (derived from IHIP-2 trial’s inclusion criteria) made it difficult to identify alternative breastfeeding discourses; socio-cultural factors for breastfeeding cessation may thus, not be predominant in the study setting.

## Conclusions

Women with low socio-economic and education backgrounds living in rural high-altitude Andean Peru identified the cultural beliefs that the quality of breast milk declined six months after childbirth and that breastfeeding caused child diarrhoea. These perceptions were rarely considered in routine medical care at local healthcare centres, resulting in negative attitudes towards health personnel. Future breastfeeding interventions in rural high-altitude Andean Peru should promote peer-counselling approaches. Introducing mother’s concerns and cultural breastfeeding interpretations into the Peruvian national programme on early child development could be a feasible and cost-effective solution. Additional research on socio-cultural factors for breastfeeding cessation are necessary to promote equity in health.

## Data Availability

The datasets used and/or analysed during the current study are available from the corresponding author on reasonable request.

## Abbreviations

COREQ: Consolidated Criteria for Reporting Qualitative Research
ECD: Early child development
IHIP-2: Integrated home-based intervention package trial 2
LMIC: Low- and middle-income countries
PNCM: Peruvian national programme on early child development
UPCH: Cayetano Heredia university
WHO: World Health Organization
